# Role of MOH as a grassroots public health manager in preparedness and response for COVID-19 pandemic in Sri Lanka

**DOI:** 10.3934/publichealth.2020048

**Published:** 2020-08-05

**Authors:** Pamila Sadeeka Adikari, KGRV Pathirathna, WKWS Kumarawansa, PD Koggalage

**Affiliations:** Ministry of Health and Indigenous Medical Services, Colombo, Sri Lanka

**Keywords:** COVID-19, medical officer of health, pandemics, prevention and control, public health

## Abstract

Pandemic transmission of COVID-19 virus warranted activation of public health responses in all countries. Public health unit system of Sri Lanka (also known as the Medical Officer of Health unit system) managed by a medical doctor with special training in public health, the Medical officer of Health (MOH), with a team of grassroots field staff who are well aware of the community and supported by a network of infrastructure. The aim of the study was to describe the managerial role of the MOH as a grassroots public health manager in the preparedness and response for COVID-19 pandemic. The research team studied the key documents communicated to MOH by the national authorities. The study revealed, national level authorities used the MOH to implement COVID-19 control and preventive decisions through their technical and managerial directives. MOH unit earned trustworthiness in the community due to their deep-rooted ground level operations. Further, MOH system possess deep understanding and extensive connectivity with the community. Therefore, implementation of rigid prevention and control measures was well facilitated within the assigned geographical public health unit area.

## 1. Introduction

COVID-19 is a viral disease thought to have been transmitted to humans through close animal-human activity. It is a coronavirus with the potential of spreading from human to human across geographical boundaries within a brief period [1]. Several exported cases reported throughout the world and the first case of COVID-19 was identified in Sri Lanka on 27 January 2020 in a foreign national. Sri Lanka rapidly mobilized her limited resources to combat the crisis. Reported number of cases and fatalities were maintained low, showing the high resilience of the government-funded public health system [2].

**Figure 1.**
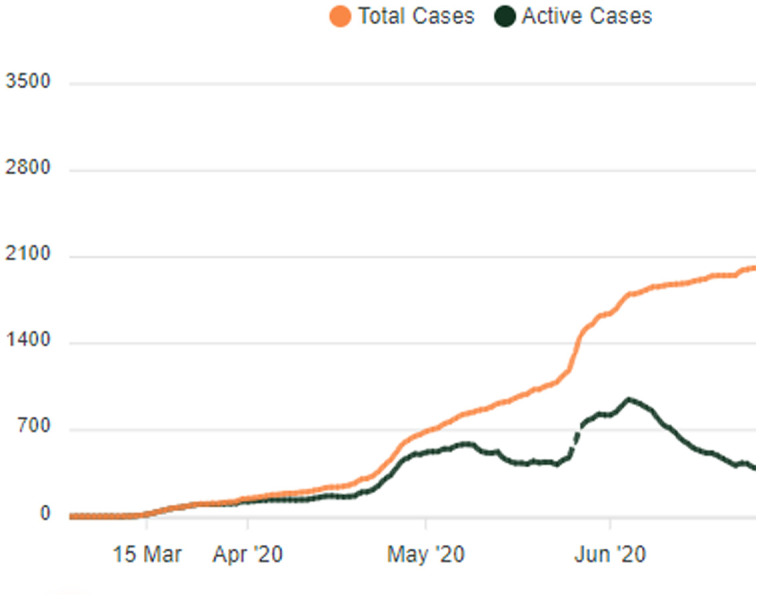
Total versus active cases reported in Sri Lanka within the first three months (Live Situational Analysis Dashboard of Sri Lanka, Health Promotion Bureau, Ministry of Health).

Total versus the active cases reported in the country within the first three months after reporting the first case of COVID-19 is displayed in Figure 1.

Sri Lanka is an island nation in the Indian Ocean with a population of 21.4 million [3]. The country's experience in successfully controlling communicable diseases such as polio, malaria, lymphatic filariasis, neonatal tetanus, vertical transmission of HIV when all her neighbours are badly affected, proves the success of the public healthcare system. Moreover, Sri Lankan tropical climatic conditions is a favourable breeding ground for several vector-borne and zoonotic diseases such as dengue, leishmaniasis and leptospirosis [4]. The country has been unaffected by several other diseases with pandemic potential such as ebola, MERS-CoV and Influenza A. The geographically defined public health system has proven to be successful in managing such outbreaks [5].

Sri Lankan primary healthcare is delivered through two distinctive modalities; curative and preventive [5]. However, most primary healthcare systems in the world are limited only to ambulatory care [6]. The preventive care system of Sri Lanka is separated into geographic subdivisions known as Medical Officer of Health (MOH) Units [5] that cater for 60,000 to 100,000 people [7]. Established first in Kalutara in the Western Province in 1926, the system now expanded to cover the whole island. MOH area can be described as the smallest administrative division in public health in Sri Lanka [8]. Internationally, only a handful of countries share a unit based preventive health system similar to Sri Lanka [6].

Sri Lanka has a robust public health system. It has achieved impressive health outcomes with greater benefits obtained per dollar spent on healthcare [9],[10]. In India, the maternal mortality ratio amounts to 400 per 100,000 live births while spending over 5% of GNP but in Sri Lanka, the ratio is 36 and the country spends only 3% of GNP on health [11]. The success behind these achievements is thought to be due to appointing MOH as the in-charge of a specific geographical area or the MOH unit. Each member of the community, according to their place of residence, is assigned to a MOH.

The MOH Unit system has been recognized as a role model to bring preventive healthcare services with greater effectiveness and efficiency [12]. The MOH manages human, physical and financial resources for the control and prevention of COVID-19 as recommended by the national technical authorities through his command on key actions and priority areas of work in preparedness and response against the pandemic as mentioned in the interim guidance by WHO [13].

Medical Officer of Health is a medical doctor and is the manager of this unit with a team of public healthcare workers who maintains preventive services at the grassroots level. Administrative and supervisory staff members are located in the MOH office. Field staff such as public health inspector (PHI), public health midwife (PHM), school dental therapist, the staff of disease control programs are located in the field [7]. Responsibilities of MOH are in par with the five core essential functions identified by WHO Europe, namely, surveillance, monitoring preparedness for response, health protection, health promotion and disease prevention [6],[14]. The technical leadership for MOH activities is given nationally by the Epidemiology Unit, Family Health Bureau, Non-communicable diseases unit and other respective preventive health programs. In the provincial level, district-level medical doctors who represent the above units guide the technical operations [5]. Besides, MOH is the administrative leader responsible for financial and supervisory actions.

The MOH of Sri Lanka plays a key role at the grassroots level of the public health system that performs preventive health activities on multidisciplinary specialities; communicable, non-communicable, maternal and child health, elderly care, occupational and school health [5],[14]. A successful public health system as described by the WHO Europe should be a multidisciplinary speciality that performs multifaceted functions and multisectoral policy actions should be in place for it to be successful [6]. MOH performs multifaceted functions on surveillance, monitoring preparedness for response, health protection, health promotion and disease prevention throughout his duties as a response for emerging and re-emerging diseases [15]. MOH led programs with collaborations of multiple stakeholders have been successful in controlling dengue, an endemic vector-borne tropical disease [16]. MOH plays a key role in public health emergencies to preserve the health of those who are affected [17]. Further, MOH is empowered by the multisectoral policy actions in Sri Lanka [18].

The authority vested by the quarantine and prevention of disease ordinance to the Director-General of Health Services, the Head of the Health Department of Sri Lanka, is delegated to the MOH in a pandemic situation [15],[19]. When the World Health Organization announced COVID-19 as a public health emergency of international concern, MOH played a key role in the control and management of COVID-19 pandemic and to prevent its spread within and outside his geographical territory. MOH provided leadership to the technical instructions informed to them through proper channels for the prevention and control of COVID-19. The tasks assigned to them in the pandemic were nothing incongruous. MOH is the focal and the backbone of the public healthcare system at the grassroots level.

**Figure 2.**
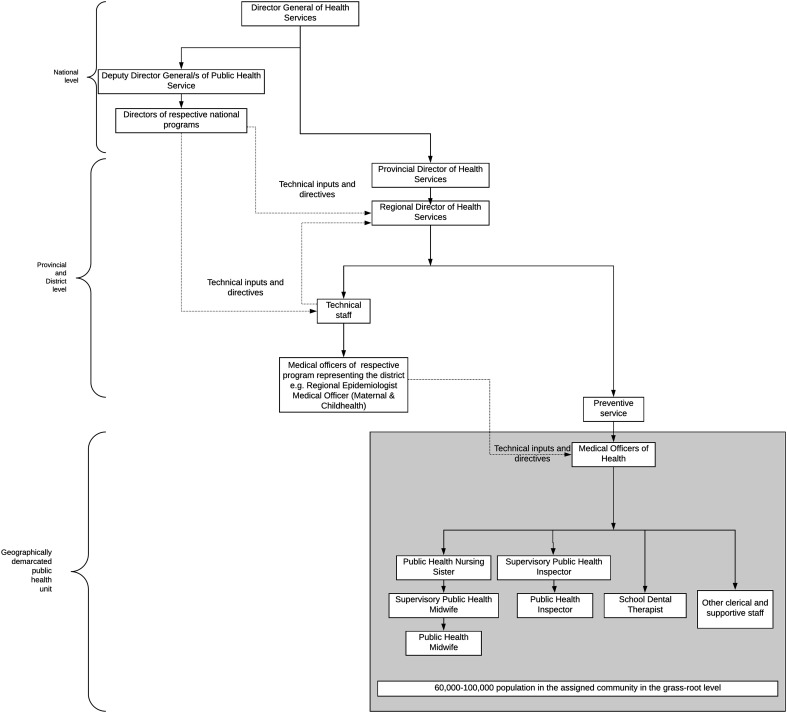
Organisational structure of Public Health Services in Sri Lanka.

MOH is the manager of resources and the public health team. The team is ready to discharge quick response within a very short notice, using their already available infrastructure and capacity for networking within the community and ability to update their knowledge and skills promptly. Each MOH unit has a network of community infrastructure that can be quickly converted to operational centres in the field such as field clinics. This transition is possible because of its unique system structure as geographical health unit. Each office has logistic facilities that can mobilize relevant resources and public health teams, into the field when the need arises. Throughout health promotion programs, MOH and his field staff maintain a high level of community engagement to achieve sustainable public health goals [20]. The organisation of public health services within the Sri Lankan health system are displayed in Figure 2.

In a highly connected world, pandemics like COVID-19 would spread at an exponentially high speed. Due to its location in the Indian Ocean, Sri Lanka engages in a significant number of international transactions. Sri Lanka's high population density of 341 per square kilometre [3] favours a rapid spread of a communicable disease like COVID-19. Being a middle-income country, current health expenditure ratio as a percentage to GDP is also 3%, a comparably low value than a developed country such as the United States of America; 17.8% in 2019 [21]. However, Sri Lanka's persistent resilience on facing COVID-19 spread might have lessons to learn from her public health system. Since 01 January 2020 to 03 June 2020, reported cases and fatalities remain 1730 and 11 respectively, showing effective public health intervention in COVID-19 control programmes which MOH played a major role in grass root level [22]. Studying the unique managerial role of MOH as the operational and technical agent at grass root level and how they ensure prompt and effective discharge of the national level guidelines and directives within his well-established public health unit in COVID-19 control activities will pave healthcare decision-makers and other countries to learn from Sri Lanka's experience.

The aim of the study was to describe the managerial role of the MOH as a grassroots public health manager in the preparedness and response for COVID-19 pandemic in a geographically defined public health unit system in Sri Lanka.

## 2. Material and methods

This is a non-systematic narrative review of COVID-19 related documents such as circulars and guidelines issued by the Ministry of Health and Indigenous Medicine of Sri Lanka within the period from 01 January 2020 to 20 May 2020. All the documents received by three convenient MOH offices during the study period were searched manually and electronically using the key words; COVID-19, pandemic, response, preparedness, prevention, novel coronavirus, (2019-nCoV) and quarantine. The list of documents was further confirmed for their suitability for inclusion into the study by verification process by senior MOHs who are currently working in public health units. The study team consisted of experts in healthcare management who have experience in both curative and preventive public healthcare systems.

The relevant documents were converted into electronic format and shared among the team members. Each member went through all the documents and electronically highlighted the relevant sections. Highlighted data was entered into a spreadsheet under the heading of key-information. Coding was done considering extracted key information phrases from the documents and information was synthesized and organized under the identified action areas by the common agreement through a series of discussions by the study group.

## 3. Results and discussion

During the COVID-19 pandemic in Sri Lanka, the MOHs performed an important role in public health response activities as a grassroots manager. His scope of work is guided and regulated by expert technical advice from national as well as provincial health authorities. Studying key information from the documents issued by the Sri Lankan health authorities to the MOHs was carried out by the study group, comprising of health management experts, to describe the unique public health role in the management of COVID-19 pandemic. The summary of the studied government documents are listed in Table 1.

**Table 1.** Summary of government documents reviewed.

Date	No	Source	Title	Key thematic areas
2020-04-17	NA	Directorate of Environmental Health, Occupational Health and Food Safety, Ministry of Health	Operational guidelines on preparedness and response for COVID-19 outbreak for work settings	Business continuity and workplace public health measures
2020-04-17	EPID/EPI/40/2018	Epidemiology Unit, Ministry of Health	Guidance on resumption of immunisation services during COVID-19 outbreak	Maintaining routine public health services
2020-03-18	FHB/MCU/COVID-19/2020	Family Health Bureau, Ministry of Health	Interim guidelines for maternal and new-born care services in hospitals during the outbreak of COVID-19 infection 18th March 2020	Case finding, contact tracing and management including quarantine
2020-03-21	NA	Epidemiology Unit, Ministry of Health	Updated interim case definition and advice on initial management	Case finding, contact tracing and management including quarantine Surveillance
2020-04-23	VER 3.1	Health Promotion Bureau, Ministry of Health	COVID-19 (new Coronavirus) Outbreak in Sri Lanka Interim Guidelines for Sri Lankan Primary Care Physicians	Case finding, contact tracing and management including quarantine
2020-04-24	NA	Director General of Health Services, Ministry of Health	Management of Accidental Discovery of Suspected COVID-19 Patient in the Hospital	Protecting field staff
2020-03-25	EPID/400/2019 NCoV	Epidemiology Unit, Ministry of Health	Maintenance of a register for healthcare workers exposed to COVID-19 at healthcare institutions	Protecting field staff
2020-01-26	DDG(PHS)1/DO2/12-9/2019/10	Deputy Director General (Public Health Service 1), Ministry of Health	Interim summary guidelines for clinical management of patients with novel coronavirus (2019-nCoV)	Risk communication Case finding, contact tracing and management including quarantine Public health measures Laboratory testing Protecting field staff
2020-03-01	NA	Ministry of Health	Provisional Clinical Practice Guidelines on COVID-19 suspected and confirmed patients	Case finding, contact tracing and management including quarantine
2020-04-02	NA	Ministry of Health, Epidemiology Unit, College of Microbiologists and WHO	Correct use of PPE to prevent spread of COVID-19 (Presentation)	Protecting field staff
2020-04-02	NA	Directorate of Environmental health, Occupational health and Food safety, Ministry of Health	Guidelines on COVID-19 preparedness for workplaces	Business continuity and workplace public health measures
2020-04-03	EPID/400/2019 n-CoV	Ministry of Health	Screening and management of healthcare workers following exposure to a confirmed/suspected case of COVID-19	Protecting field staff
2020-03-04	DDG (PHS1)/DO2/ 12-9/ 2019/10	Ministry of Health	Revision of interim summary guidelines for clinical management of patients with novel coronavirus	Surveillance
2020-05-04	EPID/400/2019 n-CoV	Ministry of Health	Guidance on carrying out RT-PCR test for COVID-19 in work settings	Surveillance Laboratory testing
2020-05-07	EPID/400/2019 n-CoV	Ministry of Health	Strengthening of COVID 19 Surveillance	Surveillance
2020-02-11	FHB/SHU/Let/2020	Ministry of Health	Guidelines for School settings	Public health measures
2020-03-31	PA/DDG PHS11/3/COVID/Gen/2020	Ministry of Health	Guideline for rational use of PPE for community health staff	Case finding, contact tracing and management including quarantine
NA	EPID/400/FORM/2019 n-CoV	Epidemiology Unit, Ministry of Health, Sri Lanka	Case investigation form for foreign and local.	Case finding, contact tracing and management including quarantine
NA	NA	Directorate of Environmental health, Occupational health and Food safety, Ministry of Health	Guidelines on COVID-19 preparedness for workplaces	Business continuity and workplace public health measures
		Epidemiology Unit, Ministry of Health	Guideline for the Home quarantine, Quarantine in non-health care settings	Risk communication Surveillance Case finding, contact tracing and management including quarantine
NA	NA	Epidemiology Unit, Ministry of Health	2019-nCoV: Early detection for outbreak response and follow up.	Surveillance
NA	NA	Epidemiology Unit, Ministry of Health	Interim guidance for Sri Lankan flights to and from China-Instructions to be given to all passengers boarding from China	Surveillance
2020-05-13	DDG(EOHFS)/13-9/2017	Director General of Health Services, Ministry of Health	Operational guidelines on preparedness and response for COVID-19 outbreak for work settings	Case finding, contact tracing and management including quarantine Business continuity and workplace public health measures
2020-05-14	FHB/EU/GOSL/COVID-19/2020	Family Health Bureau, Ministry of Health	Interim guidelines for resuming field RMNCAYH care services during the outbreak of COVID-l9 infection (Date: 13th May 2020)	Maintaining routine public health services
2020-03-15	NA	Epidemiology Unit, Ministry of Health	Guidance for workplace preparedness for COVID-19	Business continuity and workplace public health measures
2020-04-15	DDG(EOHFS)/13/11/2020	Directorate of Environmental health, Occupational health and Food safety, Ministry of Health	Guideline for facilitating operation of export-oriented manufacturing and processing factories during COVID-19 outbreak	Business continuity and workplace public health measures
2020-03-17	FHB/MCU/COVID-19/2020	Family Health Bureau, Ministry of Health	Interim guidelines for field maternal and childcare services during the outbreak of COVID-19 infection (Date 2020 March 17)	Maintaining routine public health services

The preventive wing of the public health system was promptly activated by the Sri Lankan Ministry of Health, to improve the preparedness and discharge responses during the pandemic.

The study revealed that the preparedness and the response role played by the MOH in grassroots level falls into following action areas; risk communication, surveillance, case finding, contact tracing and management including quarantine, public health measures, laboratory testing, case management, maintaining routine public health services, protecting field staff, business continuity/workplace public health measures and acting as a technical agent for national public health programs within his geographical unit for staff training.

1. Risk communication: Educating and communicating actively with the public to enhance their level of community engagement for increase their preparedness and response against the spread of the pandemic are one of the priority areas of work that is performed by the public health system of any country [13]. This function has been addressed at the earliest stage of the pandemic. During the COVID-19 pandemic, Health Promotion Bureau of Sri Lanka acted as the national focal point to risk communication. MOH acted as the grassroots manager for implementing the guidance within their public health unit. Their familiarity, developed connections, trustworthiness with the community and their awareness of community values and beliefs assisted effective risk communication process.

2. Surveillance: The purpose of surveillance is aiding rapid detection of suspected cases and to implement control measures to contain outbreak [23]. Surveillance is one of the highly emphasised areas in the pandemic, observing 7 correspondence documents within 60 days from national-level authorities. Considering the exponential speed of the spread on the infection, all surveillance methods had to be scaled up to be geographically comprehensive. Sri Lankan public health system with 347 geographically demarcated administrative areas as MOH units [24] would have been an opportunity for the health decision-makers to enhance surveillance for COVID-19 and to limit the spread of disease. Surveillance activities were expedited because the MOH being a qualified medical doctor with special public health training [5], and further, this was facilitated by the extensive community network that the MOH has developed through his field staff. Above mentioned surveillance activities included reporting real-time symptomatic patient, reporting contact tracing, random testing and reporting.

3. Case finding, contact tracing and management including quarantine: WHO advise enhancing contact tracing and management comprehensively across the country to break the transmission chain of the disease [13]. It is evident that, throughout the pandemic, this activity has commenced early and continually upgraded as case definitions are refined [25]. From the starting of the pandemic, the greatest number of national-level directives as circular or guideline has been released. Although the scope of the activities considered here are included in MOH normal duties under prevention and control of communicable disease, during the pandemic this action theme was more highlighted. Case finding, contact tracing and management including quarantine procedures such as self-isolation for symptomatic patients, self-isolation for contact communities (they were monitored by MOH), quarantine of risk communities within their geographical locations such as villages, lanes, slums with the help of police and military. The communities with lack of compliance and high-risk behaviours were evacuated from their residence and quarantined at high secured quarantine centres were more effectively achieved by the MOH due to his capability to reach the grassroots level community. In addition to that, in early stages of the disease transmission MOH and his staff involved in collection of swabs for RT-PCR testing in field level for symptomatic patients. As pandemic progressed within the community and with establishment of laboratory facilities within the country covering all the provinces, MOH was assigned to perform random testing in high risk areas. These rigid containment measures could effectively implemented due to the deep understanding of the social, cultural and economic status of the community. Further his field staff were more aware of the targeted population.

4. Public health measures: The highly communicable nature of COVID-19 virus from human to human warranted three major actions to be taken by the affected communities; hand hygiene, respiratory etiquette, social distancing and recommendation of universal facemask usage for the community [13],[26]. Also, they played an integral part when returning to normalcy. Public health teams had to spread the message to the community as well as the MOH unit field staff to prevent the spread of the virus. MOH led programs has been communicating messages containing public health measures throughout their battle with communicable diseases [27]. The Epidemiology Unit, Ministry of Health, Sri Lanka, which acts as the national level technical focal point of controlling communicable diseases, had a robust mechanism of delivering directives on public health measures through the MOH led public health team at a grassroots level during the COVID-19 pandemic. Public health measures were smoothly and effectively implemented because of the nature of his role as a collaborator, coordinator with external parties such as police, divisional secretary, schools, transport sector and community leaders [5]. Further his vested law enforcement power through the quarantine act enhanced above mentioned collaborative effort to implement public health measures [15].

5. Laboratory testing: Testing individuals following exposure to a patient with COVID-19, a contact of a COVID-19 patient or criteria of a suspected case definition were tested by RT-PCR test as a confirmatory test for diagnosis. During early disease transmission authorities did not recommend RT-PCR test as a screening procedure as PCR can still be negative and the patient may harbour the virus during the incubation period. With the progression of the disease and establishment of laboratory services, RT-PCR tests were randomly performed on high risk groups in repeated intervals. Due to the late antibody response, the health authorities not recommended serological tests as a screening procedure [26]. Although it is a difficult task, MOH and field staff are well experienced and skilled to implement similar screening programs in community-level such as lymphatic filariasis [28]. Therefore, national authorities utilized MOH as a grassroots manager to efficiently implement laboratory screening program that strongly linked with surveillance of cases in the community. In the early stage of disease transmission, number of RT-PCR tests performed in the island was very limited due to lack of facilities. However, with the strong government involvement several test facilities established covering all provinces. At the early stage average number of tests performed was 1075 per day and later it increased up to 4000 tests per day [29].

6. Case management: In the COVID-19 pandemic in Sri Lanka asymptomatic contact with positive travel history, a suspected case of COVID-19 with positive contact history and symptoms and immediately following up of a patient with COVID-19 after discharge was managed at the community level by the MOH [26]. Mentioned cases were home quarantined or admitted to the nearest acute care isolation hospital or followed up through public health system following registering at MOH office respectively [26]. The MOH was updated on evolving case definitions throughout by the national focal points via their district and provincial heads. This task was speeded up effectively considering his high connectivity with technical authorities as well as with the community through the field staff.

7. Maintaining routine public health services: Some routine functions of MOH such as maternal, well-baby and immunisation clinics were temporarily suspended due to travel restrictions imposed in the country to prevent disease transmission [30]. Resuming functions during return to normalcy, staff need to use a surgical facemask, setting up of triage counter to screen for respiratory diseases on the arrival of clients, both staff and clinic attendees following strict public health measures and limiting participants who attend the clinic. As the grassroots public health manager, the MOH has to improve the preparedness and provide leadership to the service delivery operations by providing consumables to practice public health and IPC measures and technical advice, training and supervision to staff whenever needed for the smooth operation of the clinic [30]. MOH being the manager and the supervisor of his public health team facilitated implementing these new administrative and operational changes effectively and communicate with community through public health field staff who were highly recognised by the community.

8. Protecting field staff: Managerial role of the MOH were further evident when he had to protect his team from the virus which is highlighted in literature as protection of frontline workers [31]. Measures such as case finding, contact tracing and management, quarantine of contacts and isolation of cases among the public health staff were taken up by the MOH as per directives by the national decision-makers. He had to oversee the IPC measures especially for the use of PPE and technical guidelines for the field staff. MOH had to maintain a register on staff members who had been exposed to a confirmed COVID-19 case and screen and management of healthcare workers in the follow-up events [32]. This important role can be considered as an important function that can boost the spirit of his team as well as ensure continuity of public health services.

9. Business continuity and workplace public health measures: Business continuity is an important domain in the societal response for COVID-19, as public health measures have negative spill over effects on the economy [33]. MOH as the grassroots health manager had to take the leadership role according to the government circulars and guidelines to restore the economy. The study revealed seven documents have been issued during the study period centralising this activity. This requirement was facilitated by the contemporary quality within the service as high connectivity, and understanding local business community through public health inspector, who is a member of MOH's field staff [34].

10. Acting as a technical agent for National public health programs within his geographical unit for staff training: MOH is the link between national-level focal points such as epidemiology unit, health promotion bureau and disease prevention programs and grassroots level field staff. In routine operations, this network has helped national-level policy and decision-makers to rapidly transfer knowledge [5],[35],[36]. During the COVID-19 pandemic, the Ministry of Health Sri Lanka used the same channel of knowledge transfer to upgrade knowledge of the field staff on public health and IPC measures and specifically routine operations of the society. To facilitate this activity MOH has well established infrastructure and knowledge and experience for training [5].

## 4. Conclusion

As a manager of a geographically defined public health unit, MOH has the power to enforce law entrusted to them by the quarantine and prevention of disease ordinance to make provision for preventing introduction and spreading of contagious or infectious diseases aiming protection of community. In a highly communicable disease like COVID-19 rigid public health measures which have direct adverse effects needed to be implemented by the governments. In addition, negative spill over effects of these measures that deemed to be implemented to control of COVID-19, had a risk of failure at deployment. But, in the Sri Lankan context, these rigid measures were efficiently and effectively implemented at the grassroots level. The study revealed, national level authorities used MOH and field staff to implement COVID-19 control and preventive decisions through their technical and managerial directives. During this effort, MOH exhibited effective and efficient operational performance. This achievement was facilitated by sound technical knowledge, a well-established network of community infrastructure, deep rooted understanding and gained of the community and high level of connectivity with the community. The managerial role of MOH in this public health unit system should be further explored for future public health reforms.
